# Changes in Inflammatory Markers Predict the Prognosis of Resected Hepatocellular Carcinoma with Child–Pugh A

**DOI:** 10.3390/curroncol29080457

**Published:** 2022-08-16

**Authors:** Jing Zhou, Daofeng Yang

**Affiliations:** Department of Infectious Diseases, Tongji Hospital, Tongji Medical College, Huazhong University of Science and Technology, Wuhan 430030, China

**Keywords:** hepatocellular carcinoma, inflammatory markers, prognosis

## Abstract

(1) Background: The reasons for changes in the inflammatory markers of patients with surgically resected hepatocellular carcinoma are unclear. We aimed to investigate the association of an inflammatory status with the prognosis of patients with hepatocellular carcinoma, who underwent surgical resection. (2) Methods: We retrospectively enrolled 91 patients with Child A hepatocellular carcinoma, who had received surgical resection, to explore the influence of preoperative inflammatory markers and postoperative changes on the prognosis. (3) Results: The platelet-to-lymphocyte ratio (PLR) and its alteration were independent prognostic factors. Patients with a low PLR had a significantly better recurrence-free survival (RFS) than those with a high PLR (1-year RFS of 88.5% versus 50.0%; 3-year RFS of 62.1% versus 25.0%, *p* = 0.038). The patients with a low PLR showed a significantly better overall survival (OS) than those with a high PLR (1-year OS of 98.9% versus 75.0%; 3-year OS of 78.2% versus 25.0%, *p* = 0.005). The patients whose PLR had increased at 6 months after operation showed a worse OS than patients whose PLR had decreased (1-year OS of 96.3% versus 98.4%; 3-year OS of 63.0% versus 79.7%, *p* = 0.048). However, neither the neutrophil-to-lymphocyte ratio nor Onodera’s prognostic nutritional index had any prognostic significance. (4) Conclusions: The PLR and its alteration are significant prognostic factors for the RFS and OS of patients with Child A hepatocellular carcinoma who had received curative surgery.

## 1. Introduction

Liver cancer ranks sixth in the incidence of cancers in the world and fourth in cancer-related mortality in the world **[1]**. Hepatocellular carcinoma (HCC) accounts for 85–90% of primary liver cancers. The overall prognosis of HCC is poor. However, HCC patients with Child–Pugh A also have a poor RFS and OS. Thus, identifying HCC patients with a high risk of recurrence and poor prognosis in time is critical to improving their outcomes.

There is a close correlation between the host’s immune status and inflammatory response and cancer [2,3]. Inflammation plays an important role in the development of cancer, including the promotion of invasion and metastasis [4,5,6]. The development of HCC is related to changes in chronic inflammation and the immune response, and the immune microenvironment also affects the prognosis of HCC patients [7]. The peripheral blood parameters RDW-CV and RDW-SD [8,9,10], certain inflammatory indicators reflecting the status of immunity, such as the neutrophil-to-lymphocyte ratio (NLR), PLR [11,12], and lymphocyte-to-monocyte ratio (LMR) [13,14,15,16,17], as well as the nutritional parameter Onodera’s prognostic nutritional index (OPNI) [18], etc., are considered to be associated with the development and prognosis of many diseases. Many clinical studies have reported that inflammatory markers are related to the prognosis of many tumors. Inflammatory markers have the advantage of simple calculation and acquisition. Similarly, there are an increasing number of studies have evaluated the role of inflammatory markers in predicting the prognosis of HCC patients, including NLR, PLR, and OPNI. However, the relationship between these parameters and the prognosis of patients with HCC is still controversial [19,20,21], and there is little research on the influence of postoperative changes on the prognosis of HCC patients.

On this basis, we conducted a retrospective study on patients with Child–Pugh A HCC after surgery so as to explore the influences of the preoperative NLR, PLR, LMR, OPNI, RDW-CV, and RDW-SD and the postoperative changes on prognosis.

## 2. Materials and Methods

### 2.1. Case Collection

We retrospectively analyzed 91 patients with primary HCC, who underwent radical surgery in Tongji Hospital of Tongji Medical College, Huazhong University of Science and Technology, from May 2017 to March 2018, including those with Child–Pugh A liver function. Patients with other tumors, transformed liver cancers, recurrent HCC, and cholangiocarcinoma were excluded. We also did not include patients with infections and other diseases that affect inflammatory markers.

### 2.2. Clinicopathologic Variables

We obtained the demographic information (including gender and age at the time of resection), medical history (including hepatitis B virus and cirrhosis), and clinicopathological features (including TNM stage, tumor number, the maximum diameter of the largest tumor, and pathological grade of the resected tumor) from the patients. We also reviewed the absolute neutrophil count, absolute lymphocyte count, absolute monocyte count, platelet count, albumin, serum α-fetoprotein (AFP), RDW-CV, and RDW-SD from the hospital database at one week before operation and six months after operation, and calculated the inflammatory markers as follows: the PLR was calculated using the absolute count of platelets to lymphocytes, the NLR was estimated by dividing the absolute neutrophil count by lymphocyte count, the LMR was estimated as the absolute count of lymphocytes to monocytes, and the OPNI was measured by 5× absolute lymphocyte count(×10^9^/L) + albumin(g/L). Additionally, the RFS and OS were documented using the electronic medical record system and telephone.

The OS was the main outcome indicator of this study, defined as the time from the beginning of the surgery to death or the final follow-up. The second outcome index of this study was the RFS, which was defined as the time from the beginning of the surgery to the recurrence, as confirmed by imaging.

### 2.3. Statistical Analysis

We used SPSS 25.0 and MedCalc 20.0 software to analyze the statistics. The receiver operating characteristic (ROC) curve was used to calculate the optimal cut-off values for the AFP, PLR, NLR, OPNI, RDW-CV, and RDW-SD. The interactions between the variables and the RFS or OS were evaluated by univariate and multivariate Cox proportional hazard analyses. The survival curves of the RFS and OS were expressed using the Kaplan–Meier method and compared by the log-rank test. The Shapiro–Wilk test was used to evaluate the normality of the continuous variables. The continuous variables that conformed to the normal distribution were expressed as the mean ± standard deviation, and the continuous variables with a skewed distribution were represented as the median (interquartile range). The categorical variables were expressed as the frequency and percentage. In our study, *p* values lower than 0.05 were considered statistically significant.

## 3. Results

### 3.1. Patient Characteristics

During the study period, a total of 91 patients met the study conditions. The baseline clinicopathological features of the patients are shown in Table 1.

### 3.2. Cut-Off Values of the Preoperative AFP, PLR, NLR, LMR, OPNI, RDW-CV, and RDW-SD for Predicting the RFS and OS

The optimal cut-off value of the AFP for predicting the RFS was 10.130 ng/mL, with an area under the curve (AUC) of 64.5%. The sensitivity and specificity were 74.4% and 52.1%, respectively. The optimal cut-off value of the NLR for predicting the RFS was 2.271, with an AUC of 54.3%. The sensitivity and specificity were 39.5% and 75.0%, respectively. The optimal cut-off value of the PLR for predicting the RFS was 228.644, with an AUC of 44.6%. The sensitivity and specificity were 7.0% and 97.9%, respectively. The optimal cut-off value of the LMR for predicting the RFS was 4.633, with an AUC of 44.8%. The sensitivity and specificity were 23.3% and 81.2%, respectively. The optimal cut-off value of the OPNI for predicting the RFS was 51.925, with an AUC of 44.8%. The sensitivity and specificity were 27.9% and 79.2%, respectively. The optimal cut-off value of the RDW-CV for predicting the RFS was 13.700, with an AUC of 62.1%. The sensitivity and specificity were 32.6% and 91.7%, respectively. The optimal cut-off value of the RDW-SD for predicting the RFS was 42.550, with an AUC of 61.7%. The sensitivity and specificity were 76.7% and 50.0%, respectively.

The optimal cut-off value of the AFP for predicting the OS was 10.535 ng/ml, with an AUC of 58.4%. The sensitivity and specificity were 75.0% and 46.3%, respectively. The optimal cut-off value of the NLR for predicting the OS was 4.191, with an AUC of 47.9%. The sensitivity and specificity were 20.8% and 95.5%, respectively. The optimal cut-off value of the PLR for predicting the OS was 302.104, with an AUC of 42.2%. The sensitivity and specificity were 8.3% and 100.0%, respectively. The optimal cut-off value of the LMR for predicting the OS was 3.785, with an AUC of 50.1%. The sensitivity and specificity were 45.8% and 67.2%, respectively. The optimal cut-off value of the OPNI for predicting the OS was 56.200, with an AUC of 40.3%. The sensitivity and specificity were 16.7% and 95.5%, respectively. The optimal cut-off value of the RDW-CV for predicting the OS was 13.250, with an AUC of 60.3%. The sensitivity and specificity were 58.3% and 67.2%, respectively. The optimal cut-off value of the RDW-SD for predicting the OS was 42.650, with an AUC of 63.7%. The sensitivity and specificity were 83.3% and 46.3%, respectively.

### 3.3. Prognostic Factors of the RFS and OS

The median duration of the follow-up was 44.73 months. At the end of the follow-up period, 73.6% of the patients were alive and 52.3% of patients were free from tumor recurrence. The 1- and 3-year OS were 98.9% and 75.8%, respectively, and the 1- and 3-year RFS were 86.8% and 60.4%, respectively. In the univariate analysis of the RFS, seven variables were identified as prognostic factors. These were the AFP, albumin, tumor diameter, vascular invasion, PLR, RDW-CV, and RDW-SD, respectively. Multivariate analyses of the RFS identified PLR ≥ 228.644 (*p* = 0.001), RDW-CV ≥ 13.700 (*p* = 0.028), and RDW-SD ≥ 42.550 (*p* = 0.038) as independent factors of a worse prognosis (Table 2). In the univariate analysis of the OS, four variables were identified as prognostic factors. There were the NLR, PLR, RDW-CV, and RDW-SD, respectively. Multivariate analyses of the OS identified PLR ≥ 302.104 (*p* = 0.006) and RDW-SD ≥ 42.650 (*p* = 0.031) as independent factors of a worse prognosis (Table 3).

**Figure 1:**
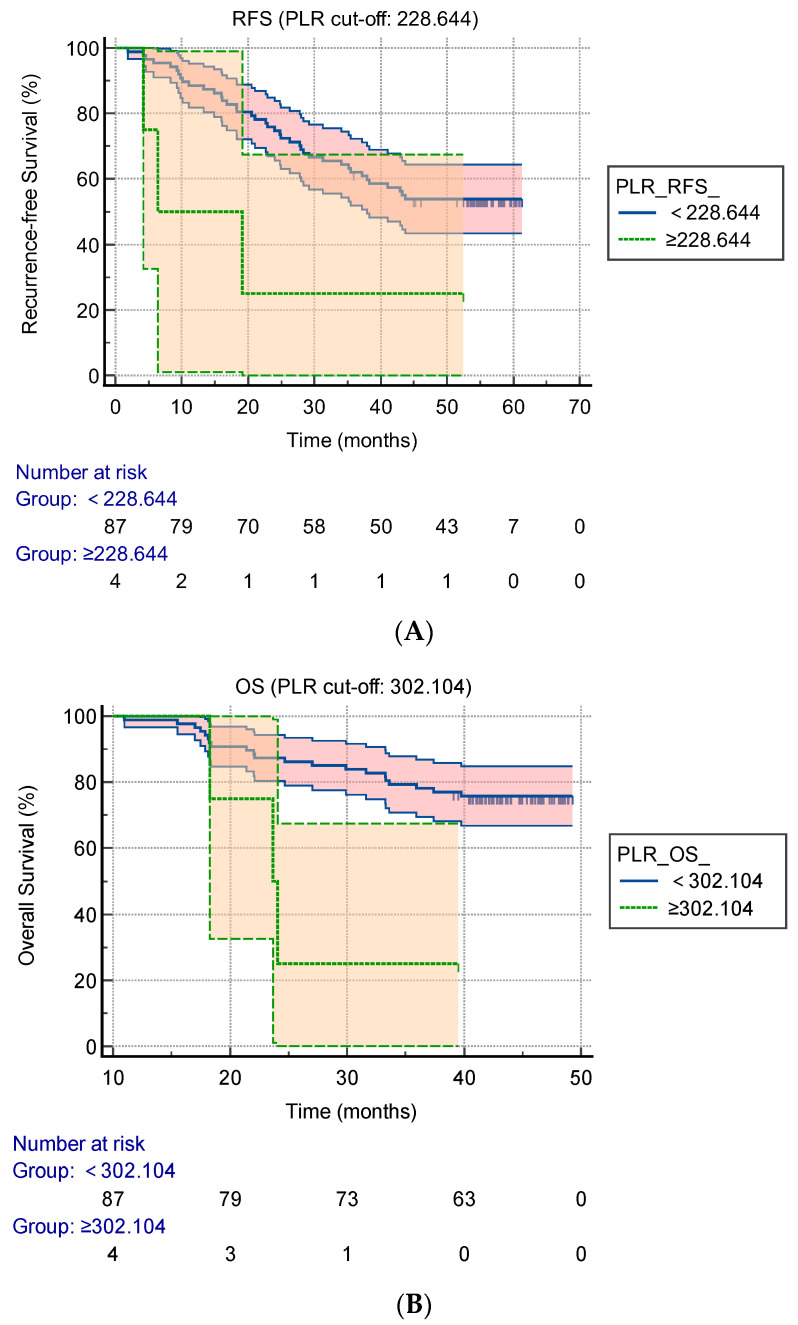
Kaplan–Meier survival plots according to the preoperative PLR. (**A**) Kaplan–Meier analysis of the RFS according to the PLR, with log-rank test (cut-off: 228.644); (**B**) Kaplan–Meier analysis of the OS according to the PLR, with log-rank test (cut-off: 302.104). PLR: platelet-to-lymphocyte ratio, RFS: recurrence-free survival, OS: overall survival.

The results of the Kaplan–Meier RFS curves with regard to the PLR (cut-off value was 228.644) are expressed as Figure 1A. The patients with a PLR of < 228.644 showed a significantly better RFS than those with a PLR of ≥ 228.644 (1-year RFS of 88.5% versus 50.0%; 3-year RFS of 62.1% versus 25.0%, *p* = 0.038). The results of Kaplan–Meier OS curves with regard to the PLR (cut-off value was 302.104) are expressed as FIGURE 1.B. The patients with a PLR of < 302.104 showed a significantly better OS than those with a PLR of ≥ 302.104 in long-term survival (1-year OS of 98.9% versus 75.0%; 3-year OS of 78.2% versus 25.0%, *p* = 0.005).

### 3.4. The Changes in the Pre-1-Week Operative and Post-6-Month Operative PLR and Their Impact on the OS

**Figure 2:**
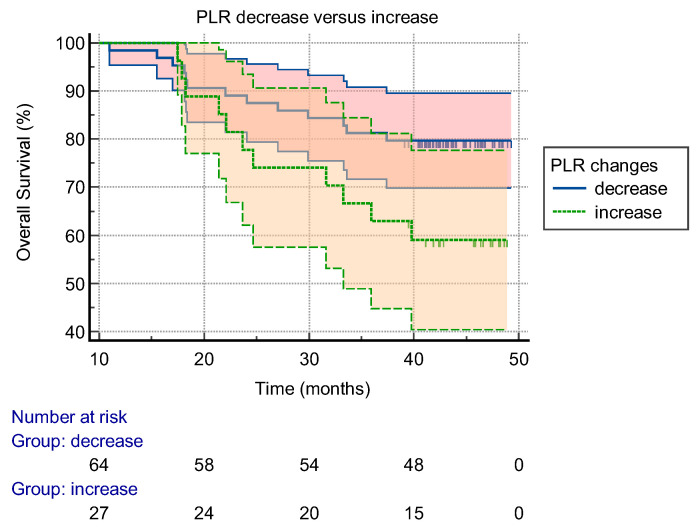
Kaplan–Meier survival plots according to the changes in PLR before and 6 months after operation. PLR: platelet-to-lymphocyte ratio.

The results of the Kaplan–Meier OS curves with the PLR changes before and 6 months after the operation are expressed in Figure 2. The patients who exhibited an increased PLR after 6 months had a worse OS than patients whose PLR had decreased (1-year OS of 96.3% versus 98.4%; 3-year OS of 63.0% versus 79.7%, *p* = 0.048). The changes in the RDW-SD had no impact on the OS. The changes in the PLR, RDW-CV, and RDW-SD were not prognostic factors of the RFS.

## 4. Discussion

Hepatocellular carcinoma, as one of the cancers with high a morbidity and mortality in China and throughout the world, is a serious threat to people’s health. At present, there are many kinds of treatment methods for patients with liver cancer, such as surgical resection, liver transplantation, chemotherapy, radiotherapy, targeted therapy, immunotherapy, and traditional Chinese medical treatments. Except for early-stage liver cancer patients, who can receive surgical resection or liver transplantation, the other methods are mostly used as rescue treatments of advanced HCC patients. Although there are various treatment methods, the recurrence rate and mortality rate of hepatocellular carcinoma are still high. Surgical resection is the main treatment for patients with early-stage liver cancer. In order to offer medical intervention to patients with a high risk of recurrence as soon as possible and reduce the recurrence rate and mortality rate of HCC, it is extremely important to use biomarkers to predict the prognosis of the postoperative patients in time, before and after surgery.

During tumor formation, it is inevitable that the body will produce inflammatory immune responses, namely cancer-associated immune responses [22], which include systemic inflammatory responses and microenvironmental inflammatory responses. HCC is no exception, and the inflammatory reaction in vivo plays an important role in the occurrence, development, and even metastasis of HCC [23]. In the occurrence and development of HCC, the inflammatory reaction is regulated by a variety of cytokines, such as interferon, tumor necrosis factor, interleukin, etc. The absolute values and ratios of neutrophils, lymphocytes, and monocytes, as the main inflammatory cells in the peripheral blood, can reflect the body’s inflammatory state to a certain extent, and the cytokines they secrete have pro-tumor and anti-tumor effects. Serum albumin is synthesized by the liver, and hypoalbuminemia usually occurs when the liver function is damaged or the body is malnourished, thus weakening the body’s immune defense ability. The RDW also reflects the state of systemic inflammation, and it has been proven to be a prognostic marker of a variety of diseases [24,25,26,27]. However, the relationship of RDW with the prognosis of HCC patients is not clear. Inflammatory biomarkers have the advantages of low cost for their identification and minimal trauma for patients, and they are simple and widely available to use. Therefore, in this study, we took neutrophils, lymphocytes, monocytes, erythrocytes, platelets, and albumin as the research objects, and calculated the NLR, PLR, LMR, and OPNI. Clinical values were taken when analyzing the blood inflammatory biomarkers to predict the prognosis in HCC patients with Child–Pugh A who underwent therapeutic hepatic resection.

First of all, we investigated the HCC patients with Child–Pugh A who underwent therapeutic hepatectomy at our hospital. The median follow-up time was 44.73 months. The 1-year RFS and OS were 86.8% and 98.9%, respectively, and the 3-year RFS and OS were 60.4% and 75.8%, respectively. Secondly, we determined that the optimal cut-off values for predicting the RFS and OS, which were 228.644 and 302.104, respectively. Then, through Cox univariate and multivariate analyses, we identified the independent predictors of the RFS and OS. A poor RFS was related to a preoperative PLR of ≥228.644, RDW-CV of ≥13.700, and an RDW-SD of ≥42.550, and the factors influencing a poor OS included a preoperative PLR of ≥302.104 and RDW-SD of ≥42.650. Overall, the preoperative levels of PLR and RDW-SD were identified as predictors of the RFS and OS in patients with Child–Pugh A hepatocellular carcinoma who underwent therapeutic hepatectomy. Therefore, we continued to collect routine blood information from the patients for the first half of the year after the operation and analyzed the influence of changes in the PLR and RDW-SD on the patients in terms of the RFS and OS. Through the Kaplan–Meier survival analysis, it was found that the 3-year OS of the patients with a decreased PLR was better than that of patients with an increased PLR in the first half-year after the operation (*p* = 0.048). 

Yang et al. have demonstrated that a high PLR is an independent predictor of a poor prognosis in HCC patients undergoing hepatic resection [28], a conclusion consistent with our findings. Wang et al. found no significant correlation among the prognosis of patients with HCC after hepatectomy [29], nor was this the case in our research. Jing et al. revealed that HCC patients with a low RDW have a relatively better prognosis [30]. From our research, we can draw a similar conclusion, namely, that a low RDW can be used as an effective predictor of a good prognosis for patients with HCC, particularly the RDW-SD, which is significantly associated with both the PFS and OS. Wang et al. proved that the OPNI can predict the prognosis of HCC patients undergoing therapeutic hepatectomy [31]. However, our research did not identify the predictive ability of the OPNI, which may be associated with the small sample size and lack of absolute representation. 

In short, from the results of our research, the preoperative PLR and RDW-SD can be identified as predictors of the RFS and OS, while change in the postoperative PLR is still a predictor of the OS. In addition, the preoperative RDW-CV can also be used as an index for predicting the RFS. Our findings indicate to clinicians that patients with a higher PLR and RDW before operation should be treated with drugs or other interventions in the early postoperative period in order to improve survival rates and reduce recurrence. However, the specific choice of postoperative treatment should also be determined according to the patient’s specific conditions and economic conditions.

There are some possible reasons why the PLR can predict the prognosis of patients with HCC. Platelets are involved in almost all the steps of cancer development [32]. Platelets can secrete transforming growth factor, interleukin, hepatocyte growth factor, and other cytokines that interact with the tumor microenvironment, which can induce the immune escape of the tumor cells and promote the formation of tumor neovascularization. Tumor cells can also activate the platelets, which can protect the tumor cells from the destructive effects of the immune system [33]. Platelets can also interact with tumor cells to promote the metastasis of tumor cells [34]. Patients with cancer who have thrombocytosis often experience adverse prognosis events [35,36]. Lymphocytes have anti-tumor effects, which can directly kill the tumor cells, but can also inhibit tumor proliferation and migration by secreting a series of cytokines, such as interferon and tumor necrosis factor [37]. The decrease in the peripheral blood lymphocytes will weaken the body’s anti-tumor ability, which can lead to tumor recurrence and progression. Thus, platelets, combined with lymphocytes, can predict the prognosis of HCC patients. Our study not only shows that a high preoperative PLR is an independent predictor of a poor prognosis in HCC patients, but also shows that the increase in the postoperative PLR remains an independent predictor of a poor prognosis.

Our study also found that a high RDW is an independent predictor of a poor prognosis in patients with HCC. The RDW refers to the change in the volume of erythrocytes, which reflect the inflammatory state of the whole body. We speculate that this may be an immune response related to cancer that acts on red blood cells, causing changes in their volume. As for its mechanisms, more basic clinical studies are needed to further clarify this.

The study has many limitations. Firstly, it is a small-scale, single-center clinical study, which makes the research results less representative, and our results require further validation by large-sample, multi-center clinical studies. Secondly, there was inevitably a selection bias when collecting the clinical information about the patients. Thirdly, the experimental data are the results of a single measurement taken one week before the operation, which may have led to numerical deviations. Fourthly, the PLR cut-off value needs further research to determine the best cut-off value to apply to clinical practice. Fifthly, this study was retrospective, and it is necessary to conduct a large-scale prospective study in order to obtain high-quality conclusions.

## Figures and Tables

**Table 1:** Baseline clinicopathological features of the patients.

Variables	All (*n* = 91)	Preoperative Parameter	Value
Gender Male Female	81 (89.0%) 10 (11.0%)	Neutrophil (×10^9^/L)	2.81 (2.05–3.77)
Age (years) <45 ≥45	27 (29.7%) 64 (70.3%)	Lymphocyte (×10^9^/L)	1.41 (1.09–1.72)
HBsAg ^a^ Positive Negative	77 (84.6%) 14 (15.4%)	Monocytes (×10^9^/L)	0.42 (0.32–0.57)
Cirrhosis Present Absent	53 (58.2%) 38 (41.8%)	Platelet (×10^9^/L)	154 (107–207)
Tumor number 1 ≥2	79 (86.8%) 12 (13.2%)	ALT ^c^ (U/L)	30 (21–45)
Tumor diameter ≤5 cm >5 cm	63 (69.2%) 28 (30.8%)	AST ^d^ (U/L)	28 (22–47)
Differentiation grade High–medium Low	58 (63.7%) 33 (36.3%)	TBIL ^e^ (umol/L)	12.40 (9.50–17.00)
Vascular invasion Yes No	23 (25.3%) 68 (74.7%)	γ-GT ^f^ (U/L)	46 (27–80)
TNM ^b^ stage I–II III–IV	73 (80.2%) 18 (19.8%)	Albumin (g/L)	40.98 ± 4.72

a: HBsAg, hepatitis B surface antigen. b: TNM, tumor node metastasis. c: ALT, alanine aminotransferase. d: AST, aspartate aminotransferase. e: total bilirubin. f: γ-glutamyl transpeptidase.

**Table 2:** Univariate and multivariate analyses of the prognostic factors of recurrence-free survival among HCC patients who received curative resection.

	Univariate		Multivariate	
	HR (95%CI)	*p* Value	HR (95%CI)	*p* Value
Gender	0.953 (0.340–2.670)	0.927		
Age (≥45 years)	1.095 (0.562–2.133)	0.789		
HBsAg ^a^ (yes)	0.930 (0.413–2.091)	0.860		
Cirrhosis (yes)	0.796 (0.431–1.467)	0.464		
Tumor number (=1)	1.271 (0.564–2.863)	0.562		
Tumor diameter (>5 cm)	2.104 (1.145–3.866)	0.017	1.737 (0.897–3.362)	0.101
Differentiation grade (I)	0.644 (0.343–1.208)	0.170		
Vascular invasion (yes)	0.523 (0.279–0.981)	0.043	0.685 (0.342–1.372)	0.286
TNM ^b^ stage (I–II)	1.660 (0.836–3.295)	0.148		
Albumin (<35 g/L)	0.392 (0.173–0.884)	0.024	0.889 (0.340–2.323)	0.810
AFP ^c^ (≥10.130 ng/mL)	2.392 (1.204–4.751)	0.013	1.986 (0.943–4.180)	0.071
NLR ^d^ (≥2.271)	1.703 (0.923–3.139)	0.088		
PLR ^e^ (≥228.644)	3.757 (1.146–12.318)	0.029	9.870 (2.573–37.861)	0.001
LMR ^f^ (≥4.633)	1.361 (0.686–2.703)	0.378		
OPNI ^g^ (≥51.925)	1.247 (0.649–2.394)	0.057		
RDW-CV ^h^ (≥13.700)	3.126 (1.642–5.949)	0.001	2.391 (1.101–5.193)	0.028
RDW-SD ^i^ (≥42.550)	2.358 (1.160–4.794)	0.018	2.305 (1.045–5.085)	0.038

a: HBsAg, hepatitis B surface antigen. b: TNM, tumor node metastasis. c: AFP, alpha-fetoprotein. d: NLR, neutrophil-to-lymphocyte ratio. e: PLR, platelet-to-lymphocyte ratio. f: LMR, lymphocyte-to-monocyte ratio. g: OPNI, Onodera’s prognostic nutritional index. h: RDW-CV, red blood cell distribution width coefficient of variation. i: RDW-SD, red blood cell distribution width standard deviation.

**Table 3:** Univariate and multivariate analyses of the prognostic factors of overall survival of HCC patients who received curative resection.

	Univariate		Multivariate	
	HR (95%CI)	*p* Value	HR (95%CI)	*p* Value
Gender	1.893 (0.647–5.542)	0.244		
Age (≥45 years)	0.873 (0.374–2.040)	0.754		
HBsAg ^a^ (yes)	0.766 (0.228–2.568)	0.666		
Cirrhosis (yes)	0.522 (0.217–1.260)	0.148		
Tumor number (=1)	0.763 (0.261–2.232)	0.621		
Tumor diameter (>5 cm)	0.459 (0.205–1.024)	0.057		
Differentiation grade (I)	2.131 (0.934–4.862)	0.072		
Vascular invasion (yes)	1.910 (0.836–4.365)	0.125		
TNM ^b^ stage (I–II)	0.655 (0.260–1.651)	0.370		
Albumin (<35 g/L)	0.385 (0.144–1.031)	0.058		
AFP ^c^ (≥10.535 ng/ml)	2.181 (0.865–5.496)	0.098		
NLR ^d^ (≥4.191)	4.712 (1.748–12.700)	0.020	2.203 (0.721–6.728)	0.166
PLR ^e^ (≥302.104)	4.894 (1.429–16.758)	0.011	9.423 (1.922–46.208)	0.006
LMR ^f^ (≥3.785)	1.542 (0.691–3.444)	0.290		
OPNI ^g^ (≥56.200)	2.583 (0.882–7.564)	0.083		
RDW-CV ^h^ (≥13.250)	2.451 (1.088–5.524)	0.031	2.014 (0.847–4.787)	0.113
RDW-SD ^i^ (≥42.650)	3.557 (1.215–10.410)	0.021	3.949 (1.134–13.748)	0.031

^a^: HBsAg, hepatitis B surface antigen. ^b^: TNM, tumor node metastasis. ^c^: AFP, alpha-fetoprotein. ^d^: NLR, neutrophil-to-lymphocyte ratio. ^e^: PLR, platelet-to-lymphocyte ratio. ^f^: LMR, lymphocyte-to-monocyte ratio. ^g^: OPNI, Onodera’s prognostic nutritional index. ^h^: RDW-CV, red blood cell distribution width coefficient of variation. ^i^: RDW-SD, red blood cell distribution width standard deviation.

## Data Availability

The details of the data can be obtained from the corresponding author upon reasonable request.
